# Early ST-segment elevation acute myocardial infarction after thrombolytic therapy for acute ischemic stroke: A case report

**DOI:** 10.1097/MD.0000000000013347

**Published:** 2018-12-14

**Authors:** Maria Mirabela Manea, Dorin Dragoş, Emanuel Stoica, Adrian Bucşa, Ioana Marinică, Sorin Tuţă

**Affiliations:** a National Institute of Neurology and Neurovascular Diseases; b “Carol Davila” University of Medicine and Pharmacy; c University Emergency Hospital Bucharest; d Institute for Cardiovascular Diseases, C.C. Iliescu, Bucharest, Romania.

**Keywords:** acute ischemic stroke, direct thrombin inhibitors, intravenous thrombolysis, STEMI

## Abstract

**Rationale:**

Acute ST-segment elevation myocardial infarction (STEMI) is a rare complication of acute ischemic stroke (AIS) during thrombolytic therapy. We report a case of STEMI occurring 40 minutes after thrombolytic therapy for AIS and discuss the possible mechanisms and therapeutic approaches.

**Patient concerns:**

A 87-year-old woman with a history of arterial hypertension was admitted for acute onset of right-sided limb weakness 2 hours before arrival at the emergency department. Forty minutes after intravenous recombinant tissue plasminogen activator (i.v. rtPA) administration for AIS, STEMI occurred (signaled by a third-degree atrioventricular block).

**Diagnoses:**

The diagnoses were AIS and STEMI. Coronary angiography confirmed right coronary artery occlusion.

**Interventions:**

Four hours after the onset of STEMI, stenting was performed, normalizing the coronary blood flow.

**Outcomes:**

The patient died 2 days thereafter because of persistent cardiogenic shock.

**Lessons:**

Our case is remarkable owing to the unusually early (<1 hour) occurrence of STEMI after i.v. rtPA administration. A third-degree atrioventricular block after thrombolysis for AIS could signal a STEMI onset. New and ongoing trials are assessing whether adjunct administration of direct thrombin inhibitors of rtPA in the first 24 hours after thrombolysis for AIS can prevent early recurrent ischemic events.

## 1 Introduction

Intravenous thrombolysis with recombinant tissue plasminogen activator (i.v. rtPA) is the first-line treatment in patients with acute ischemic stroke (AIS) within 3 to 4.5 hours from onset. The well-known complications of i.v. rtPA for AIS are hemorrhagic events or allergic reactions. Acute myocardial infarction is a rare complication of administration of i.v. rtPA as a thrombolytic agent in patients with AIS, with an unknown incidence. Only a few cases are reported in the literature, and the mechanisms are still unclear. The shortest time lag reported in the literature between rtPA treatment for AIS and the onset of ST-segment elevation myocardial infarction (STEMI) is 2 hours.^[1–6]^ The reporting authors posited that fragmentation of an existing left ventricular thrombus is 1 of the mechanisms of STEMI in the wake of thrombolytic therapy. The mortality in such patients is high, although no definitive conclusions can be drawn given the scarcity of relevant reported cases. Table 1 is a summary of the reported cases of STEMI after i.v. rtPA treatment for AIS.

**Table 1:**
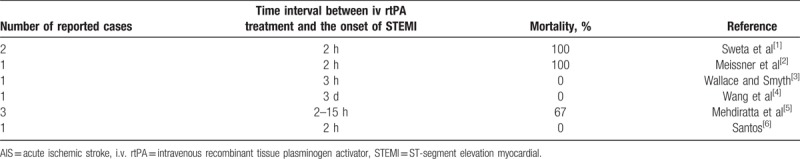
Cases of STEMI after i.v. rtPA treatment for AIS reported in literature.

## 2 Presenting concerns

A 87-year-old woman with a history of arterial hypertension was admitted to our clinic for acute onset of a right-sided limb weakness 2 hours before presentation to our emergency department.

Informed written consent was obtained from the patient for publication of this case report and accompanying images.

## 3 Clinical findings

Clinical examination results were normal, except for a slightly increased arterial blood pressure of 150/90 mm Hg. Neurological examination revealed moderate expressive aphasia, central facial paresis, and right-sided hemiparesis. The National Institutes of Health Stroke Scale (NIHSS) score was 12.

## 4 Diagnostic focus

**Figure 1:**
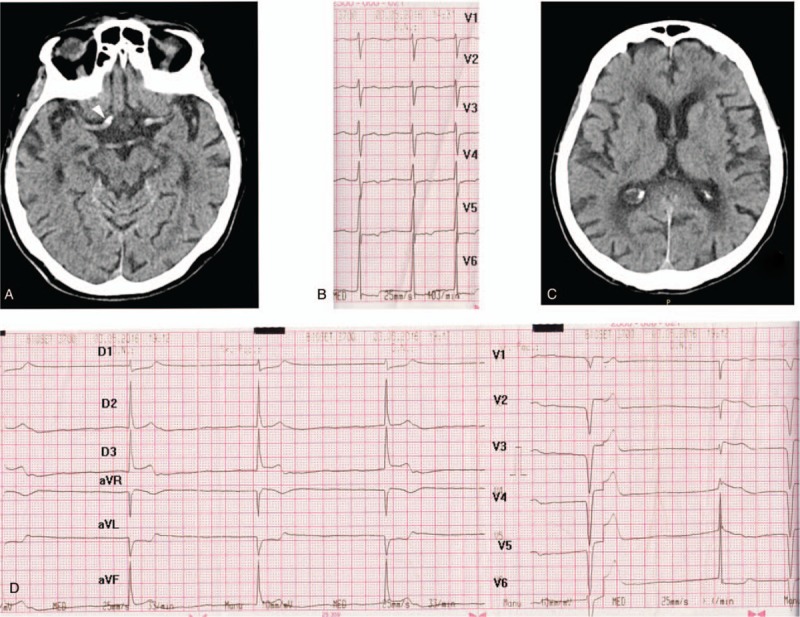
(A) Right middle cerebral artery calcification (arrowhead). (B) Atrial flutter. (C) Grade 3 Fazekas leukoaraiosis, cerebral atrophy. (D) Ventricular premature beats and third degree atrioventricular block, Hisian rhythm, ST-segment elevation in DII, DIII, aVF, V2, V3, V4 leads.

Cerebral computed tomography (CT) revealed right middle cerebral and basilar artery calcifications (Fig. 1A), grade 3 Fazekas leukoaraiosis, cerebral atrophy (Fig. 1C), and an Alberta Stroke Program Early CT Score (ASPECTS) of 10. The electrocardiography (ECG) performed at admission revealed an atrial flutter (AFL) and a heart rate of 62 bpm (Fig. 1B). The troponin T level at admission was within its normal range (0.01 ng/mL).

## 5 Therapeutic focus and assessment

**Figure 2:**
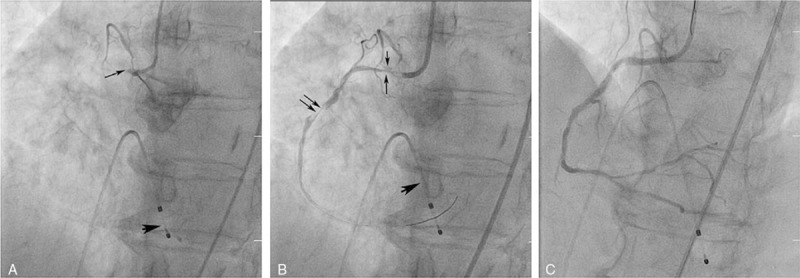
(A) Left anterior oblique (LAO) projection of the right coronary artery (RCA) depicting occlusion at the origin (thin arrow) with no contrast material penetration to the distal vessel. Temporary pacing wire in the right ventricle (arrow head). (B) Left anterior oblique (LAO) projection of the right coronary artery (RCA) after balloon predilation: dissection area (downward pointing arrow) and stenosis (upward pointing arrow) in the first segment of the right coronary artery (RCA), stenosis suggestive of thrombus in the second segment of the RCA (double arrow). (C) Left anterior oblique (LAO) projection of the right coronary artery (RCA) at the end of the procedure: satisfactory repermeabilization of the vessel.

Forty-five minutes after admission, the patient was thrombolysed with i.v. rtPA (6.3 mg bolus and 56.7 mg for 1 hour) in accordance with the current guideline,^[7]^ which led to some neurological amelioration (a decrease in NIHSS score to 8). However, 40 minutes after rtPA infusion, the patient developed significant chest pain. Continuous ECG monitoring revealed a sudden onset of severe bradycardia (38/min) due to third-degree atrioventricular block, most probably associated with Hisian rhythm and ventricular premature beats (Fig. 1D), and a decrease in blood pressure to <80 mm Hg. The i.v. administrations of inotropes and atropine were started. Soon afterward (within 10–15 minutes), ST-segment elevation was observed in the inferior (DII, DIII, and aVF) and anteroseptal (V2, V3, and V4) leads (Fig. 1D). Consequently, the patient was transferred to the cardiology department of another hospital, where transthoracic echocardiography (TTE) revealed hypokinesia of the inferior interventricular septum, and inferior and inferolateral walls, and a moderately to severely reduced (40%) ejection fraction, but no intracardiac thrombus. Coronary angiography (Fig. 2) confirmed the occlusion of the right coronary artery (thrombolysis in myocardial infarction [TIMI] flow 0) and severe stenoses (>70%) on the other coronary branches (left circumflex, left marginal, and left anterior descending branches). Given the compromised cardiac function, an aggressive and potentially life-saving therapeutic approach was chosen to restore the permeability of the occluded coronary artery. At first, thrombus aspiration was attempted (4 hours after the onset of STEMI), but failed to extract any thrombotic material. Subsequently, stenting was performed and blood flow was re-established to normal (TIMI flow III) in the previously occluded coronary artery. Thereafter, antithrombotic therapy, including heparin, and dual antiplatelet therapy (clopidogrel and aspirin) were started in accordance with the established guidelines for acute coronary syndrome,^[8]^ taking into account the potential risk of cerebral hemorrhagic complications.

## 6 Outcomes

Unfortunately, the patient died 2 days after because of persistent cardiogenic shock.

## 7 Discussion

Our case is remarkable by at least 2 features. First is the unusual occurrence of STEMI (signaled by a third-degree atrioventricular block) after i.v. rtPA administration for AIS. The normal ECG and troponin values at admission were strong indicators that the STEMI did not precede stroke onset. Second is the very early onset (<1 hour) after thrombolytic therapy. The earliest onset of myocardial infarction after fibrinolytic treatment for AIS that was reported in a few published relevant cases is 2 hours.^[1–6]^

Some possible pathogenic mechanisms have been suggested for the development of STEMI immediately after thrombolytic therapy for AIS. One is cardioembolic STEMI due to fragmentation of a pre-existent intracardiac thrombus induced by thrombolytic treatment. In a large study,^[9]^ the prevalence of coronary artery embolism was 2.9%, with atrial fibrillation (AF) as the most common cause (73% of cases) and multivessel embolism occurring in 15% of the patients. AF was associated with an ≈2-fold increased risk of MI in other studies, partly because of the embolic risk, and also because of an inflammation-associated prothrombotic state.^[10]^ Our patient had AFL, but this is frequently associated with atrial fibrillation; this could have also induced a cardioembolic stroke, although the severe stenoses afflicting multiple coronary vessels could have been associated with similar lesions in the patient's cerebral arteries. However, given the short time of observation and the severe condition of the patient, an evaluation of the cervical-cerebral artery status was not performed. On the contrary, in 1 study, an intracardiac thrombus was detected in 2.7% of patients with AIS who underwent i.v. rtPA, but showed no evidence of consequent prompt embolization, which suggests that the thrombolytic treatment is not necessarily associated with precocious embolic recurrence.^[11]^ The TTE conducted in the cardiology department did not reveal evidence of a thrombus in our patient. Of course, a TTE with contrast enhancement, or a transesophageal examination, would have been more reliable in thrombi detection,^[12]^ but it was not performed in our patient, given the tight timeline imposed by the gravity of the patient's state. The second possible mechanism of STEMI is local thrombosis on an unstable atheroma plaque in a stenotic coronary artery. Coronary angiography demonstrated severe stenosis (>70%) in several coronary branches and complete occlusion of the right coronary artery. An unexpected prothrombotic status has been recently posited to arise immediately after rtPA therapy, and this could further promote coronary thrombosis. The free plasmin formed as a result of the systemic lytic state can activate contact factors, factor V, and possibly prothrombin; the thrombin bound to fibrin is progressively exposed as the clot undergoes lysis and has the potential to locally activate platelets and accelerate coagulation.^[13]^ This prothrombotic status supposedly induced by rtPA administration could partly explain the myocardial reinfarction that occurs in 3% to 6% of patients, despite successful coronary thrombolysis^[14]^ or arterial reocclusion with early neurological deterioration in 14% to 34% of thrombolysed stroke patients.^[14,15]^ Although alteplase has a short plasma half-life of about 4 to 6 minutes,^[16]^ therapeutic guidelines recommend against antithrombotic treatment in the first 24 hours after i.v. thrombolysis for fear of increased risk of bleeding.^[17,18]^ Nevertheless, a systematic review concluded that the absence of antiplatelet therapy and cerebral artery stenosis are associated with the early neurological deterioration in 13.8% of i.v. rtPA-treated stroke patients, and in only 20% of these, the neurological degradation was caused by symptomatic intracerebral hemorrhage (SICH).^[19]^ On the contrary, in the ARTIS trial,^[20]^ early administration of aspirin in patients with AIS treated with alteplase not only failed to improve the outcome at 3 months but also increased the risk of SICH. As excessive thrombin generation after i.v. rtPA clot lysis is an earlier stage than platelet activation and thrombin bound to fibrin derivatives is susceptible to inactivation by direct thrombin inhibitors,^[13]^ administration of these kinds of agents could be more appropriate than aspirin as an adjunctive therapy to rtPA for preventing rethrombosis of coronary or cerebral arteries after successful recanalization. This therapeutic direction gained more support after the argatroban with recombinant tissue plasminogen activator for acute stroke-2 trial,^[21]^ which proved that stroke patients treated with rtPA and adjunctive argatroban—a direct thrombin inhibitor—had no increased risk of SICH. Within the limit of a small study, adjunctive rtPA plus argatroban was superior to rtPA alone. In another small study, CLEAR-Full Dose Regimen (CLEAR-FDR),^[22]^ the incidence rate of SICH in patients with AIS treated with a combination of full-dose rtPA and eptifibatide was within the range of the historical rate of cerebral hemorrhage in rtPA trials.

## 8 Conclusions

The STEMI could be a rare complication of stroke thrombolysis with an early onset after i.v. rtPA administration (<1 hour in our patient). A third-degree atrioventricular block after thrombolysis for AIS could signal the onset of a STEMI, and if confirmed, it should trigger rapid initiation of coronary revascularization procedures. Some recent small-scale trials investigated the association of administration of some antithrombotics in the first 24 hours after thrombolysis as adjuncts to rtPA treatment and the prevention of some early lethal or disabling events, such as reocclusion of the coronary or cerebral arteries after successful recanalization.

## Author contributions

**Conceptualization:** Maria Mirabela Manea, Sorin Tuta.

**Data curation:** Maria Mirabela Manea, Dorin Dragos, Sorin Tuta.

**Formal analysis:** Dorin Dragos, Sorin Tuta.

**Investigation:** Maria Mirabela Manea, Emanuel Stoica, Adrian Bucsa, Ioana Marinica.

**Methodology:** Maria Mirabela Manea, Dorin Dragos, Sorin Tuta.

**Project administration:** Maria Mirabela Manea, Sorin Tuta.

**Supervision:** Sorin Tuta.

**Validation:** Dorin Dragos, Sorin Tuta.

**Visualization:** Dorin Dragos, Sorin Tuta.

**Writing – original draft:** Maria Mirabela Manea.

**Writing – review & editing:** Maria Mirabela Manea, Dorin Dragos, Sorin Tuta.
